# From trash to treasure: the role of bacterial extracellular vesicles in gut health and disease

**DOI:** 10.3389/fimmu.2023.1274295

**Published:** 2023-09-29

**Authors:** Desen Sun, Pan Chen, Yang Xi, Jinghao Sheng

**Affiliations:** ^1^ Department of Biochemistry and Molecular Biology, Zhejiang Key Laboratory of Pathophysiology, School of Basic Medical Sciences, Health Science Center, Ningbo University, Ningbo, China; ^2^ Affiliated Hangzhou First People’s Hospital, Zhejiang University School of Medicine, Hangzhou, China

**Keywords:** bacterial extracellular vesicles, outer membranes vesicles, gut health, intestinal barriers, immune barrier, inflammatory bowel disease, cancer, gut disease treatments

## Abstract

Bacterial extracellular vesicles (BEVs) have emerged as critical factors involved in gut health regulation, transcending their traditional roles as byproducts of bacterial metabolism. These vesicles function as cargo carriers and contribute to various aspects of intestinal homeostasis, including microbial balance, antimicrobial peptide secretion, physical barrier integrity, and immune system activation. Therefore, any imbalance in BEV production can cause several gut-related issues including intestinal infection, inflammatory bowel disease, metabolic dysregulation, and even cancer. BEVs derived from beneficial or commensal bacteria can act as potent immune regulators and have been implicated in maintaining gut health. They also show promise for future clinical applications in vaccine development and tumor immunotherapy. This review examines the multifaceted role of BEVs in gut health and disease, and also delves into future research directions and potential applications.

## Introduction

The gut is an intricate and dynamic ecosystem that plays a pivotal role in human health and disease (1–3). Housing approximately 100 trillion organisms, the influence of the gut microbiota extends beyond simple digestion (1, 4). They can shape metabolic functions, influence epithelial barrier integrity, regulate immune responses (5–7). These microorganisms interact with host cells in numerous ways, from direct cellular adhesion or invasion to the release of cell wall components and the secretion of metabolically functional products (8–10). Emerging research recognizes that bacteria can modulate gut health via producing bacterial extracellular vesicles (BEVs) (11).

BEVs represent a class of cellular products secreted by both gram-negative and positive bacteria (12–14). These vesicles are usually 20 – 400 nm in diameter and have a bilayer lipid membrane structure with a similar composition to that of the parent membrane (15). Protected by the membrane, BEVs encapsulate various substances including virulence factors, proteins, nucleic acids, and lipids (13). The primary function of BEVs are considered as an excretion system for the disposal of unwanted metabolites and misfolded proteins (16). Moreover, BEVs are found to function as signal and material transmission tools that mediate bacteria-bacteria and bacteria-host interactions (13, 15). BEVs can aid bacteria in nutrient acquisition, resistance to antibiotics or antimicrobial peptides (AMPs), and elimination of specific microbes (17). Meanwhile, BEVs can deliver virulence factors and toxins to host cells, thereby disrupt barrier integrity, induce inflammation, and even promote carcinogenesis (18). Nevertheless, the BEVs from certain beneficial or commensal bacteria are contribute to host health maintenance by triggering a host defence response or immune activation (11).

In this review, we consolidate the published evidence demonstrating the impact of BEVs on gut health, particularly their role in regulating the integrity and function of the intestinal barrier. We also highlight the significant roles of BEVs in various gut diseases, including infection, inflammatory bowel disease (IBD), gut-related metabolic diseases, and gastrointestinal tumors. We discuss the limitations of current research on BEVs in the gut, while concurrently exploring their potential therapeutic applications in gut disease treatment.

## Biogenesis and types of BEVs in the gut

The gastrointestinal tract harbors a dynamic and symbiotic microbial ecosystem (19). These microorganisms exhibit remarkable metabolic abilities and continuously secrete BEVs into the lumen. Recent studies have reported a significant concentration of 8 × 10^12^ BEVs per milliliter in a solution containing 20 g of stool resuspended in 100 ml phosphate-buffered saline (20). Typically, these BEVs are classified into outer membrane vesicles (OMVs) and cytoplasmic membrane vesicles (CMVs), based on their constituent parts and unique biogenesis pathways (13).

**Figure 1:**
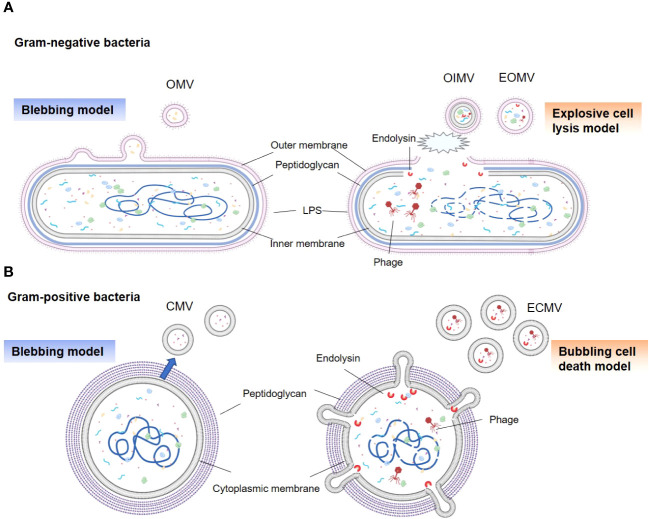
Types and generation models of BEVs. **(A)** Gram-negative bacteria can release OMVs by blebbing of the outer membrane (left panel). Vesicles produced by explosive cell lysis are named explosive outer membrane vesicles (EOMVs) and outer-inner membrane vesicles (OIMVs), which are triggered by phage-derived endolysin that degrades the peptidoglycan layer (right panel). EOMVs and OIMVs randomly contain cytoplasmic components, while OMVs don’t directly package cytoplasmic components. **(B)** Gram-positive bacteria can secret cytoplasmic membrane vesicles (CMVs) (left panel). Stress induced Gram-positive bacteria lysis, named “bubbling cell death”, can lead to the release of ECMV (right panel).

The ability of gram-negative bacteria to secrete membrane vesicles originating from their outer membranes, termed as OMVs, was discovered over fifty years ago (14, 21). Subsequent research has revealed that gram-negative bacteria generate several types of BEVs under various conditions, including OMVs, outer inner membrane vesicles (OIMVs), and explosive outer membrane vesicles (EOMVs) (14, 22). Traditional OMVs are formed through a process known as “blebbing” (or the non-lytic route), resulting in a vesicle encapsulated in a single membrane bilayer (**Figure 1A**) (22). OMV generation is attributed to several mechanisms, including reduced outer membrane-peptidoglycan connection linkages, increased membrane curvature, increased periplasmic pressure, and flagellar rotation (22–24). Additionally, during genotoxic stress, gram-negative bacteria may utilize explosive cell lysis (or the lytic route) to produce OIMVs and EOMVs (13, 22). The prominent feature of these vesicles is both OIMVs and EOMVs contain many cytoplasmic components; moreover, OIMVs have two membrane bilayers, derived from the outer and inner membranes. (**Figure 1A**) (25).

Although enveloped in a dense peptidoglycan layer, gram-positive bacteria have evolved to generate their own types of vesicles, termed as CMVs (14). Similar to OMVs, these vesicles are encased in a lipid bilayer derived from the cytoplasmic membrane of the parent bacteria and exhibit a comparable size range (**Figure 1B**). The precise process underlying CMVs biogenesis remains elusive; however, a series of pivotal steps have been identified (26–28). First is cytoplasmic membrane budding, prompted by the accumulation of specific phospholipids in the outer leaflet of the membrane (26). Next is the formation and release of CMVs from the plasma; this is influenced by lipoprotein content reduction, which increases membrane fluidity, and accumulation of phenol-soluble modulins, which disrupt membranes due to their surfactant-like properties and amphipathic helical structure (26, 29). The final step is the passage of CMV through the cell wall. This process is facilitated by peptidoglycan-degrading enzymes (27, 29). In addition, explosive CMVs (ECMVs) can be formed in gram-positive bacteria via “bubbling cell death”, which is similar to EOMV biogenesis (13). In this process, the release of CMVs under SOS response-inducing conditions is facilitated via prophage-derived endolysins (**Figure 1B**) (13, 30). However, the comprehensive elucidation of CMVs biogenesis in gram-positive bacteria remains unclear.

In addition, evidence suggests that BEV generation is accurately regulated. Recent studies on *Salmonella enterica* have indicated that the production of OMVs is upregulated by its PhoPQ system when attacked by host innate immunity (31). Antibiotic-induced oxidative stress in *S. aureus* triggers CMV production via increasing permeability of the peptidoglycan layer. Genetic regulation of vesiculation has also been investigated, with disruptions in gene encoding factor σ B (sigB) in *Listeria monocytogenes* (*L. monocytogenes*) (32) or the two-component system CovRS in *Streptococcus pyogenes* (33) resulting in altered CMVs production, which indicates a regulatory role in vesicle biogenesis. Furthermore, the cargos contained in OMVs are rigorously controlled (34, 35). The lipoprotein composition between the outer membrane of *Bacteroides thetaiotaomicron* and its OMVs were found to be significantly different (36). Moreover, a study showed that the exposure of *Pseudomonas aeruginosa* (*P. aeruginosa*) to the epoxide epibromohydrin resulted in the significant upregulation of the epoxide hydrolase (Cif) and outer membrane protein OprF in its OMVs (37).

## Role of BEVs in gut homeostasis

**Figure 2:**
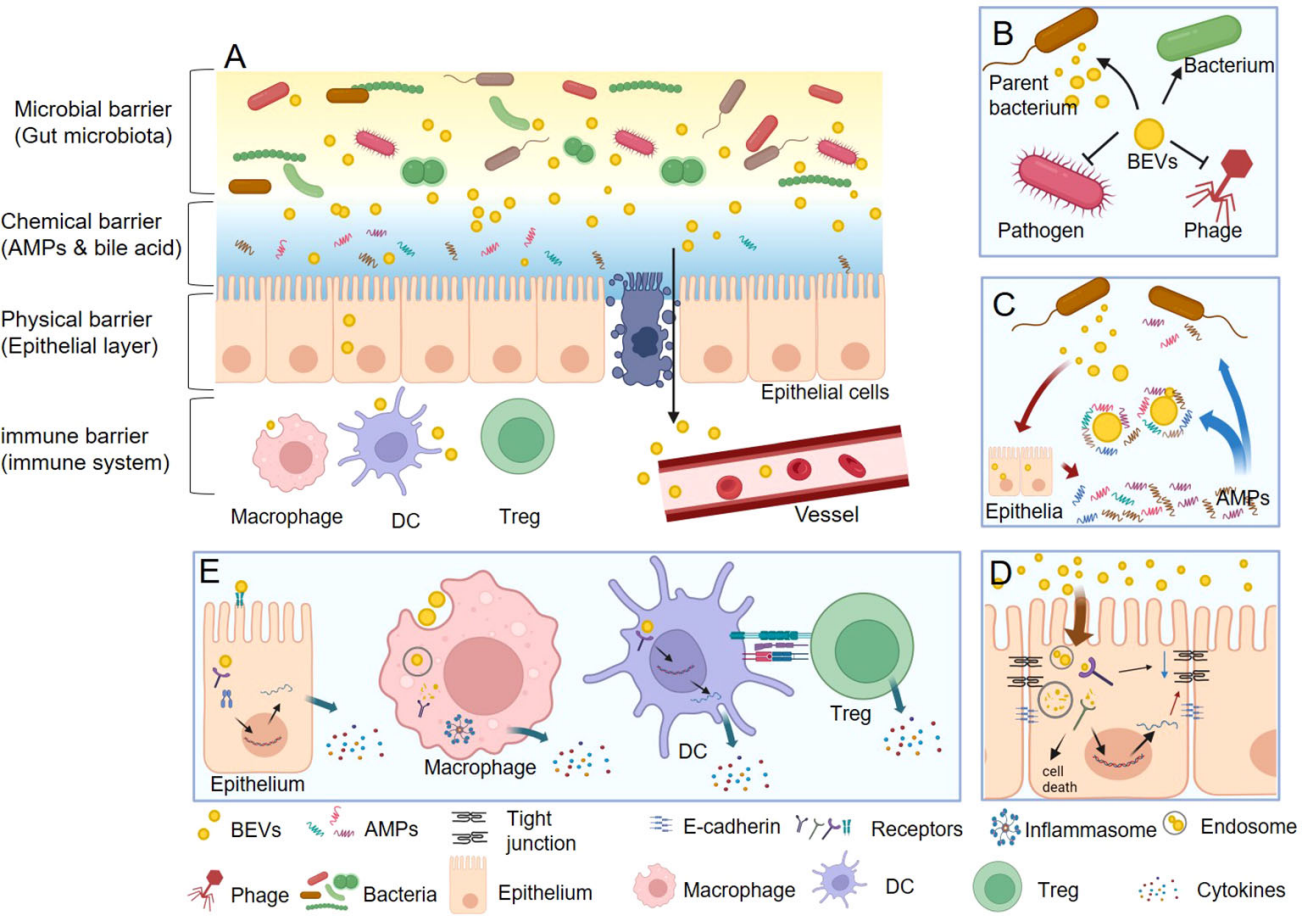
The functions of BEVs in gut homeostasis. **(A)** BEVs regulate gut health by interacting with microbial, chemical, physical, and immune barriers. **(B)** In microbial barrier, BEVs can promote the survival of their parent bacterium or other bacteria, protect against phage infection, and kill competitor species. **(C)** In chemical barrier, BEVs can neutralize the function of AMPs. Nevertheless, some BEVs also act as stimulators that induce the intestine to express more AMPs thus enhance the chemical barrier. **(D)** In physical barrier, BEVs can damage the integrality of epithelial barrier via reducing the tight junction protein and E-cadherin, or causing epithelial cell death. On the contrary, the BEVs from some beneficial bacteria could enhance the physical barrier function. **(E)** In immune barrier, BEVs could stimulate epithelial cell to secret cytokines through both cell surface receptors (such as TLR4) and inter intracellular receptors (such as NOD1). Macrophage can directly recognize and uptake BEVs and then activate inflammasome and secret cytokines. DCs can detect the polysaccharide (PSA) from OMV then result to promote the differentiation Tregs and the anti-inflammatory cytokine IL-10.

Gut homeostasis is fundamentally reliant on an intact barrier function composed of microbial, chemical, physical, and immune barriers that work together to form a defense line from the lumen to the basal layer (38–41). BEVs, which are products of gut commensal bacteria (42), serve as key messengers and regulators in this environment. They facilitate a range of interactions with the gut barrier that contribute to the maintenance of gut health (**Figure 2**).

### Microbial barrier

The healthy gut microbiota is referred to as the microbial barrier, and comprises various species of commensal intestinal bacteria (43). These species either compete or cooperate to establish a balanced microbial community (43), which is critical for resisting the colonization, growth, and invasion of pathogenic microorganisms (44). BEVs can modulate the equilibrium of the gut microbiota in several ways (**Figure 2B**). First, BEVs promote the survival of their parent bacterium or other bacteria. For instance, *P. aeruginosa* OMVs carry many *Pseudomonas* quinolone signals, which can bind iron, an essential element for bacterial viability, and bring it to the outer membrane of the parent bacterium via fusion (18). Similarly, OMVs from *Akkermansia muciniphila* (*A. muciniphila*) can restore the disturbed balance of gut microbiota via selectively promoting the proliferation of beneficial bacteria through membrane fusion (45). Second, bacteria release BEVs as a defense mechanism against phage infections. For example, Manning et al. reported that the co-incubation of OMVs collected from *Escherichia coli* (*E. coli*) and T4 bacteriophages resulted in a significant reduction in the active phage number (46). Similarly, Reyes-Robles et al. found that *Vibrio cholerae* (*V. cholerae*) secreted OMVs carrying phage receptors as a defense mechanism that conferred protection against phage predation (47). Finally, BEVs function as tools to eliminate other bacteria. Li et al. reported that OMVs from 15 strains of gram-negative bacteria, including many commensal or pathogenic gut bacteria, such as *Enterobacter*, *Escherichia*, *Morganella*, *Salmonella*, and *Shigella* strains, could lyse many gram-positive and gram-negative cultures. Peptidoglycan hydrolases associated with BEVs are thought to account for bacterial lysis (48). Growing evidence has supported the antimicrobial functions of BEVs. For instance, OMVs from *P. aeruginosa* can kill competitor species such as *S. aureus* via peptidoglycan hydrolases, antimicrobial 4-hydroxy-3-methyl-2-(2-non-enyl)-quinoline, and rhamnolipid (49). OMVs from *Lysobacter* and *Myxococcus* contain a toxic mixture of bioactive compounds and lytic enzymes capable of killing the surrounding microbes (50).

### Chemical barrier

The intestinal chemical barrier is composed of AMPs and other antibacterial substances, such as bile acids (41), and inhibits growth of certain bacteria and segregates intestinal bacteria from intestinal epithelial cells. Several studies have suggested that BEVs can disrupt the function of the chemical barrier (**Figure 2C**). Nakayama-Imaohji et al. reported that a *Bacteroides fragilis* (*B. fragilis*) strain with hypervesiculating mutants (which release more OMVs) showed higher resistance to treatment with AMPs, such as LL-37 and defensin-2 (51). Similarly, Urashima et al. found that the outer membrane protein T, which was specifically enriched in the OMVs of enterohemorrhagic *E. coli* (EHEC), broke down LL-37 and inhibited its antimicrobial activity, thereby enhancing EHEC survival and adaptation to the host gut environment (52). Moreover, the exposure of *P. aeruginosa* to lysozyme significantly enhanced OMVs release (by approximately 100-fold) (53). Analogously, *in vitro* studies have shown that *E. coli* upregulates OMVs secretion upon encountering AMPs, and the addition of *E. coli* OMVs has been demonstrated to increase bacterial survival *in vitro* when challenged with antibiotics, such as Polymyxin B and colistin (54). All in all, BEVs can digest or neutralize AMPs, potentially weakening the chemical barrier function.

Contrarily, some evidences indicated that BEVs act as stimulators, inducing the intestine to increase AMP expression, thereby enhancing the chemical barrier (**Figure 2C**). For instance, Kaparakis et al. discovered that OMVs from *P. aeruginosa* and *Helicobacter pylori* (*H. pylori*), which contained peptidoglycans, could induce epithelial cells to express human β-defensins (HBD), such as HBD2 and HBD3 (55). *Lactobacillus* derived CMVs have also been reported to stimulate the expression of the AMP REG3G, a c-type lectin, thus promoting the chemical barrier of the gastrointestinal tract and providing protection against pathogens (56). OMVs released from *A. muciniphila* were recently reported to stimulate goblet cells to produce mucus (45), which resisted the adhesion and stimulation of pathogenic bacteria to gut epithelial cells. These findings suggest that some BEVs can stimulate intestinal cells, leading to increased AMP and mucus production, thereby enhancing the chemical barrier.

### Physical barrier

The intestinal epithelial barrier, a physical partition separating the body’s internal environment from the lumen, is composed of a single layer of epithelial cells interconnected via tight junction proteins, such as occludin, claudins, and zonula occludens (39, 57). Although this physical barrier effectively limits the intrusion of most harmful substances, BEVs have been shown to internalize or permeate it (58). BEVs penetrate non-phagocytic host cells via five primary mechanisms: clathrin-mediated endocytosis, caveolin-mediated endocytosis, lipid raft-mediated endocytosis, macropinocytosis, and membrane fusion (17, 59). BEVs can concurrently utilize one or more pathways to infiltrate host cells, depending on their size and components. For example, OMVs derived from *H. pylori* were found to enter epithelial cells via four different mechanisms (55, 60, 61).

Once internalized, BEVs traverse the endolysosomal pathway and are subsequently degraded in lysosomes or autophagosomes (62); however, recent studies suggest that some BEVs can escape degradation and deliver their cargos into cells. Bielaszewska et al. demonstrated that after the OMVs of EHEC O157 were internalized in early endosomes through a process reliant on dynamin-dependent endocytosis, virulence factors, including Shiga toxin 2a (Stx2a), cytolethal distending toxin V (CdtV), and EHEC hemolysin, were separately transported from the vesicles via intracellular trafficking (63). Although the precise mechanisms of BEVs internalization and cargo transport remain unclear, BEVs are surmised act as a significant cargo delivery system to intestinal epithelial cells, influencing the function and integrity of the physical barrier of the gut.

Evidence suggests that some gut pathogenic bacteria can damage the intestinal barrier via BEVs (**Figure 2D**). Upon internalization in human intestinal epithelial cells, gram-negative bacterial OMVs release lipopolysaccharides (LPS) into the cytosol (64), facilitated by sorting nexin 10 (SNX10), which activates caspase-5. This leads to Lyn phosphorylation, subsequently down-regulating E-cadherin expression and impairing the intestinal barrier (64). EHEC O157 OMVs can disrupt the barrier through two pathways: the release of hemolysin, which increases mitochondrial permeability and triggers apoptosis (65), and the discharge of CdtV-B, which causes DNA damage and induces apoptosis (66). Moreover, OMVs of the pathogen *Fusobacterium nucleatum* (*F. nucleatum*) can activate the FADD-RIPK1-cCASP-3 signaling pathway, decreasing ZO-1 protein and increasing apoptosis, thereby damaging the epithelial barrier (67). Similarly, *Campylobacter jejuni* releases OMVs containing toxins that harm cellular DNA and impair the intestinal barrier (68–70). *V. cholerae* OMVs carry active proteases that induce apoptosis or necrosis, causing epithelial barrier loss during infection (71). Finally, Enterotoxigenic *B. fragilis* releases OMVs along with *B. fragilis* toxin, which disrupt the intestinal barrier via cleaving E-cadherin and affecting the zonula adherens and tight junctions in the intestinal epithelium (72).

Despite their disruptive potential, BEVs do not always impair the intestinal epithelial barrier (**Figure 2D**). The probiotic *E. coli* Nissle 1917 and commensal ECOR63 enhance barrier function via increasing tight junction protein expression (73, 74). Furthermore, OMVs produced by *E. coli* C25, a commensal bacterium, trigger a moderate release of the proinflammatory interleukin 8 (IL-8) and stimulate the transcriptional upregulation of Toll-like receptors (TLRs) in intestinal epithelial cell lines, subsequently enhancing the barrier function of epithelial cells and inhibiting bacterial internalization (75). Similarly, OMVs released from *A. muciniphila* help maintain the integrity of the intestinal barrier via penetrating the intestinal epithelial cells and boosting the expression of tight junction proteins and mucus (45, 76).

### Immune barrier

The gut immune barrier, primarily comprising immune cells including macrophages, dendritic cells (DCs), lymphocytes, mast cells, and natural killer cells, resides in the lamina propria or Peyer’s patch, situated beneath the physical barrier (77). They can gather information from the intestinal epithelial cells which produce a range of immunoregulatory signals (78). Furthermore, they directly recognize and accept certain bacterial components that permeate this barrier (79).

BEVs can stimulate intestinal epithelial cells to secrete various cytokines and chemokines that play pivotal roles in modulating intestinal immune functions (**Figure 2E**). For instance, *F. nucleatum* releases OMVs that stimulate epithelial cells, thereby increasing the activation of p-ERK, p-CREB, and NF-κB signaling pathways. This activation subsequently upregulates proinflammatory cytokines, including tumor necrosis factor, keratinocyte chemoattractant, IL-6, interferon (IFN)-γ, and monocyte chemoattractant protein (MCP)-1 (55). Thapa et al. analyzed the effect of BEVs derived from 32 different gut bacteria (26 gram-negative and six gram-positive bacteria) on intestinal epithelial cells. Their findings revealed that BEVs could induce species-specific immune responses in these cells. OMVs from gram-negative bacteria were found to trigger a stronger proinflammatory response than CMVs from gram-positive bacteria. A large proportion of the BEVs induced a significant increase in CCL20, IL-8, and CXCL1 levels in epithelial cell lines. Their research also identified LPS as the dominant proinflammatory bacterial effector that activated the caspase- and RIPK2-dependent pathways (80). OMVs can stimulate immune responses via cell surface and intracellular receptors in epithelial cells. For example, EHEC O157 OMVs induce IL-8 production in human intestinal epithelial cells via stimulating TLR4 and TLR5 (cell surface receptors), thus activating the nuclear factor NF-κB (81). In addition, these OMVs can deliver peptidoglycan into the host cell cytosol, thereby inducing innate immune responses through a NOD1 (intracellular receptor)/NF-κB dependent, but TLR-independent, mechanism (55, 59).

BEVs can also directly engage with intestinal immune cells (59), particularly macrophages, which play vital roles in the immune barrier (**Figure 2E**). Research shows that macrophages can uptake gram-negative OMVs via clathrin-mediated endocytosis. LPS from these OMVs can escape from early endosomes into the cytosol, triggering the caspase-11-dependent release of IL-1β and cell death in a dose-dependent manner (82). Previous studies found that guanylate-binding proteins recognized LPS, bound to the OMV surface, and mediated activation of the caspase-11 non-canonical inflammasome (83). Similarly, Bitto et al. reported that OMVs from *P. aeruginosa* directly activated the inflammasome in macrophages (84), which were dependent on caspase-5, a human homolog of murine caspase-11, highlighting another pathway of activation of immune responses in mice and humans via OMVs (83). Moreover, gram-positive bacteria can also initiate an immune response in macrophages via signaling pathways that differ significantly from those used by OMVs. Wang et al. discovered that *S. aureus* released CMVs that interacted with TLR2, thereby activating the NLRP3 inflammasome via potassium efflux. This led to the recruitment of apoptosis-associated speck-like protein containing a caspase recruitment domain and caspase-1 activation, resulting in the cellular release of mature cytokines IL-1β and IL-18 and the induction of pyroptosis (85). Conversely, CMVs from *Pediococcus pentosaceus* demonstrated potent anti-inflammatory properties. These CMVs facilitate the differentiation of bone marrow precursors into myeloid-derived suppressor-like cells and promote M2 macrophage polarization *in vitro* and *in vivo* (85, 86).

DCs are phagocytes and antigen-presenting cells that regulate the activation of adaptive immune responses, particularly T-helper and regulatory T (Tregs) cells (**Figure 2E**) (87). Shen et al. and Chu et al. demonstrated that DCs could detect polysaccharide found in *B. fragilis* OMVs via TLR2, which then activated growth arrest and DNA damage-inducible protein (Gadd45a) signaling, resulting in increased proliferation of Tregs and secretion of the anti-inflammatory cytokine IL-10 (88). This immune response process protected mice from severe experimental colitis (89). However, deficiencies in ATG16L1 or NOD2, two genes associated with IBD, disrupt DC-Treg cell interactions, thereby obstructing the protective function of *B. fragilis* OMVs (89). Additionally, *E. coli* OMVs induce DCs to generate T-helper cell responses in a strain-specific manner. Non-pathogenic *E. coli* strains, *E. coli* Nissle 1917 (probiotic) and ECOR63 (commensal), trigger increased secretion of Th1 polarizing cytokines (IFN-γ and IL-12) from DCs. Conversely, OMVs from ECOR12 (commensal) or ECOR53 (pathogenic) stimulate the production of higher levels of Treg-related cytokines (IL-10 and TGF-β). Despite the differences between strains, all OMVs enhance the secretion of Th17/Th22 priming cytokines (IL-6, IL-23, tumor necrosis factor-α, and IL-1β) (58).

What’s more, BEVs can access Peyer’s patches and then directly interact and activate the immune cells. Wang et al. found *A. muciniphila* OMVs are able to enter Peyer’s patches after direct delivery into the intestinal lumen, and induce higher production of immune active DCs with CD80 expression (45). Consequently, with the help of activated DCs, the productions of CD69^+^ B cells and IgA^+^ plasma cells along with total B cells are significantly augmented, thereby increasing intestinal IgA concentration (45, 90). This process is believed to reduce the relative abundance of harmful pathogens in the gut microbiota.

In conclusion, the influence of BEVs on intestinal barrier regulation is complex, with some enhancing barrier function, and others contributing to its impairment. This highlights the intricate interplay between the gut microbiota and their multifaceted effects on human health and diseases.

## BEVs and gut related diseases

Considering the significant roles of BEVs in maintaining gut homeostasis, investigation of their possible involvement in the onset and progression of gut-related diseases is appropriate. Current research indicates a key role of BEVs in various diseases related to the gut. These conditions include infections, IBD, metabolic disorders, and cancer (**Table 1**). BEVs, with their diverse biological functions and intricate interactions with host cells, may be pivotal in the pathogenesis of these conditions. Details of the specific roles of BEVs in each of these disease categories are discussed in the following sections.

**Table 1:** The function of BEVs in gut related disease.

Disease	Beneficial or harmful	Parent bacteria	Effective component	Target barrier layer	Influence	Reference
Gut infections	Harmful	Enterohemorrhagic *E. coli*	OmpT protease	Chemical barrier	Breaking down gut AMPs	(52)
		*P. aeruginosa*	Phospholipid bilayer	Chemical barrier	Absorbing and neutralizing AMPs	(53)
		*E. coli*	Phospholipid bilayer	Chemical barrier	Absorbing and neutralizing AMPs	(54)
		*P. aeruginosa*	Biofilm matrix-associated proteins	Physical barrier	Helping bacteria cope with stressful host environments by facilitating biofilm formation	(91)
		*V. cholerae*	Cholera toxin	Physical barrier	Delivering cholera toxin to epithelial cell and up-regulating cAMP	(92)
		*L. monocytogenes*	LLO and PI-PLC	Physical, immune barrier	Delivering a concentrated and varied toxin cargo to host cells.	(93)
	Beneficial	Gram-negative bacteria	LPS	Immune barrier	Eliciting caspase-11-mediated inflammatory reaction and helping the host to promote pathogens clearance	(82)
		*E. coli* C25	Unidentified	Physical barrier	Inhibiting the internalization of the parent bacterium	(75)
		Universal gut bacteria	Unidentified	Immune barrier	Stimulating immune cell to release of pro-inflammatory cytokines and promote an antiviral response	(94)
		Universal gut bacteria	DNA	Immune barrier	Activating the cGAS-STING-IFN-I dependent pathway to protect against RNA virus	(95)
		*L. monocytogenes*	sRNAs rli32	Immune barrier	Increase type I IFN expression in RIG-I-dependent manner	(96)
IBD	Harmful	Gram-negative bacteria	LPS	Physical barrier	Down-regulating E-cadherin expression	(64)
		*F. nucleatum*	Unidentified	Physical barrier	Activating RIPK1 and RIPK3 inducing epithelial necroptosis	(67)
				Immune barrier	Activating TLR4 and promoting pro-inflammatory cytokine production and leading to increased immune cell infiltration	(67)
	Beneficial	*E. coli* Nissle 1917	Unidentified	Physical barrier	Up-regulating tight junction proteins ZO-1, ZO-2, and claudin-14	(73)
		*B. fragilis*	Polysaccharide	Immune barrier	Interacting with DC cells and causeing immune tolerance	(88)
		*A. muciniphila*	Unidentified	Microbial, physical, and immune barrier	Restoring disturbed balance of the gut microbiota, maintaining the integrity of the intestinal barrier, activating B cells and DCs	(45)
Metabolism disease	Harmful	*P. panacis*	Unidentified	–	Blocking the insulin signaling pathway	(97)
	Beneficial	*A. muciniphila*	Unidentified	Physical barrier	Ameliorateing HFD-induced intestinal barrier dysfunction	(98)
Gastrointestinal Cancer	Harmful	*E. coli* MG1655	Ile-tRF-5X	–	Promoting the expression of the MAK3K4 gene, enhancing cell proliferation	(99)
		*F. nucleatum*	Unidentified	Physical and immune barrier	Inducing IL-8 expression and reducing E-cadherin and cadherin-1 gene expression	(100–102)
	Beneficial	Gram-negative bacteria	Unidentified	–	OMVs specifically targeted and accumulated in tumor tissues of syngeneic mouse colonic tumor model, subsequently triggering the production of antitumor cytokines	(103)
		*A. muciniphila*	Unidentified	Immune barrier	Enhance PD-1–based immunotherapy of CRC in a mouse model	(45)

### Infections

Numerous studies have highlighted the role of gut bacteria in exploiting BEVs to infect and harm hosts. BEVs can neutralize AMP activation, potentially undermining the effectiveness of the host chemical barriers and enhancing their susceptibility to pathogenic infections (53, 54). Furthermore, BEVs can foster the formation of biofilms and complex microbial communities, which are implicated in gastrointestinal infections and other diseases (22, 104, 105). A significant proportion of biofilm matrix-associated proteins originate from BEVs such as OMVs produced by *P. aeruginosa* (91). Several intestinal pathogens employ BEVs as vehicles for delivering toxins to gut cells during infection. *V. cholerae*, a noninvasive gram-negative pathogen, causes cholera via colonizing the small intestine and releasing a potent enterotoxin called cholera toxin (CT). Chatterjee et al. discovered that *V. cholerae* OMVs carried copious amounts of CT and could be internalized by intestinal epithelial cells, subsequently increasing cyclic adenosine monophosphate levels in a ganglioside GM1 (CT receptor)-dependent manner (92). Similarly, *L. monocytogenes*, a gram-positive intracellular pathogen, utilizes CMVs to release toxins, including listeriolysin O and phosphatidylinositol-specific phospholipase C, which cause mammalian cytotoxicity (93).

However, not all BEV effects on infections are harmful. Evidence suggests that BEVs can bolster gut defense against bacterial infections. During these infections, OMVs deliver LPS to macrophages, inducing a caspase-11-mediated inflammatory reaction that aids the host in pathogen clearance (82). Patten et al. observed that pre-incubation of intestinal epithelial cells with *E. coli* C25-derived OMVs impeded the internalization of the parent bacterium. They suggested that this was due to the mild proinflammatory response induced by OMVs in epithelial cells, which enhanced their ability to combat infection (75).

Moreover, BEVs are critical mediators in immune training and fortifying antiviral defenses. Bhar et al. found that co-inoculation of BEVs with murine norovirus led to the enhanced production and release of proinflammatory cytokines in macrophages, suggesting the potential role of BEVs in promoting an antiviral response (94). Erttmann et al. reported that gut microbiota depletion lowered systemic tonic IFN-I levels and antiviral priming, rendering the mice more susceptible to systemic viral infections. They found that the gut microbiota released DNA-containing BEVs that could permeate the intestinal barrier and circulate in the blood, delivering foreign DNA to distal host cells, thereby activating the cGAS-STING-IFN-I-dependent pathway to protect against RNA viruses (95). Additionally, Frantz et al. identified a specific small RNA (sRNA), rli32, partially derived from *L. monocytogenes* CMVs, that could infiltrate mammalian cell lines and increase IFN-I expression in a RIG-I-dependent manner (96).

### IBD

**Figure 3:**
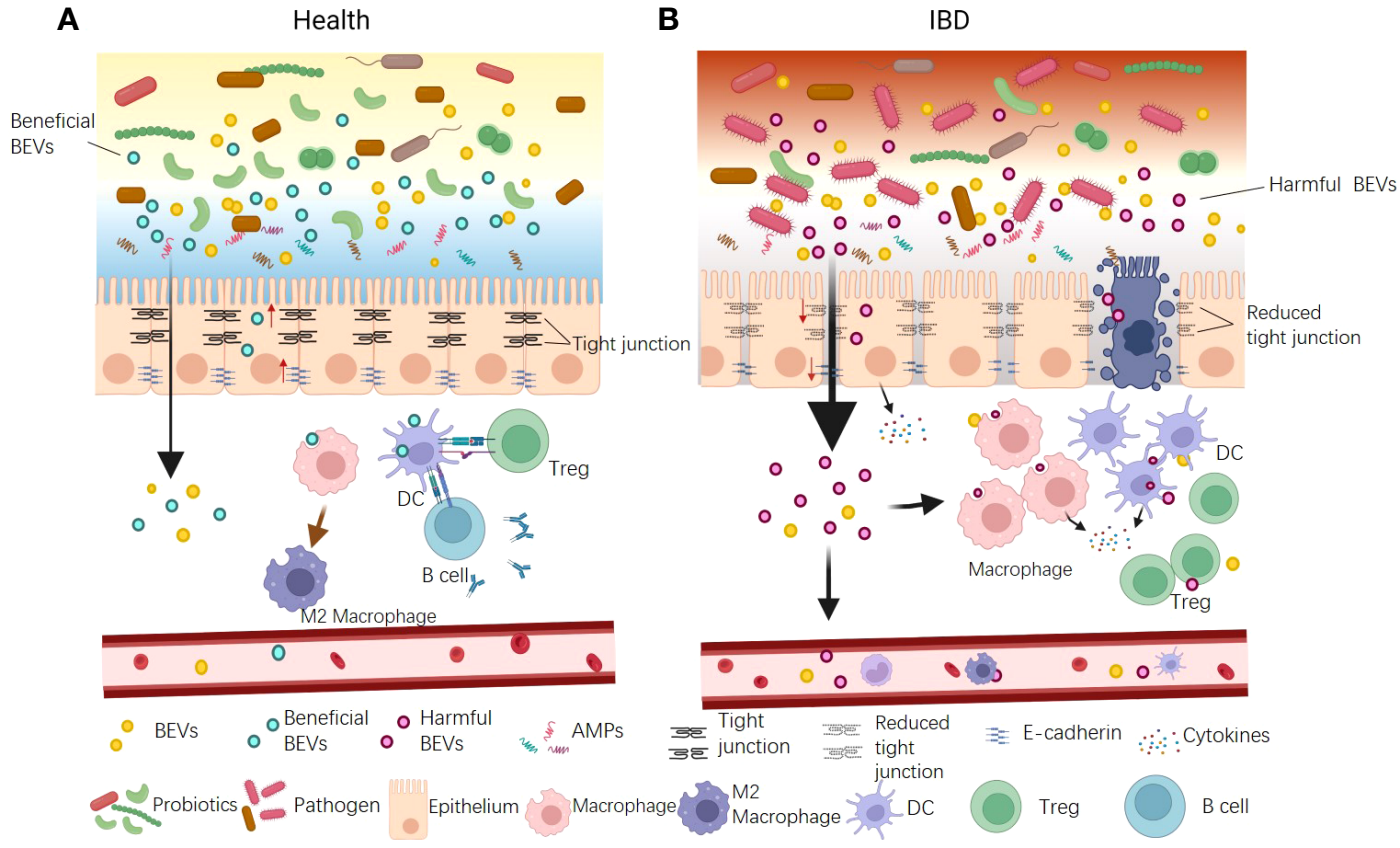
The dual functions of BEVs in IBD pathogenesis. **(A)** Some beneficial OMVs play roles in inhibiting colitis. The mechanisms include: ① enhancing gut physical barrier integrity by up-regulating tight junction proteins; ② interacting with DCs and increasing production of Tregs and the anti-inflammatory cytokine IL-10; ③ promoting anti-inflammation M2 macrophage polarization. ④ activating B cells to product mucosal immunoglobulin A. **(B)** The BEVs from pathogens can promote IBD. The mechanisms include: ① causing intestinal barrier dysfunction by down-regulating tight junction proteins and E-cadherin and inducing epithelial cell death; ② promoting pro-inflammatory cytokine production and leading to increased immune cell infiltration. In addition, BEVs could enter the blood system and stimulate peripheral blood mononuclear cells to aggravate systemic immune activation.

Numerous studies have suggested that gut barrier dysfunction can exacerbate IBD progression. Current research indicates that BEVs contribute to IBD via damaging both physical and immune barriers, particularly the epithelial and immune cells (**Figure 3A**). The increased proportion of gram-negative bacteria observed in patients with IBD typically release excessive OMVs laden with LPS (64). These OMVs infiltrate epithelial cells and their LPS translocate into the cytosol, instigating immune reactions, downregulating E-cadherin expression, and causing intestinal barrier dysfunction (64). Tulkens et al. clinically investigated and revealed a significant correlation between the levels of BEV-associated LPS in the plasma and impaired barrier integrity in patients with intestinal mucositis, including IBD (20, 106). As a result, bacterial BEVs significantly stimulate peripheral blood mononuclear cells to secrete proinflammatory cytokines such as IL-6, IL-8, MCP-1, and macrophage inflammatory protein-1α. Specific OMVs released by gut pathogens are also associated with IBD (20). Liu et al. reported that *F. nucleatum* OMVs significantly exacerbated dextran sulfate sodium (DSS)-induced colitis symptoms in mice via activating receptor-interacting protein kinases 1 and 3 and inducing epithelial necroptosis. This process resulted in significant epithelial barrier loss and oxidative stress-related damage (67). Engevik et al. supported this result and, in addition, they found that *F. nucleatum* OMVs also activated TLR4 and downstream targets signal-regulated kinase, cAMP response element binding Protein, and NF-κB, thereby promoting proinflammatory cytokine production and leading to increased immune cell infiltration (107).

Conversely, several studies have reported the protective role of BEVs against IBD (**Figure 3B**). The probiotic *E. coli* Nissle 1917 enhances gut physical barrier integrity via upregulating the tight junction proteins ZO-1, ZO-2, and claudin-14, thereby attenuating DSS colitis in mice (73). The commensal bacterium *B. fragilis* secretes OMVs that interact with DCs, triggering immune tolerance and thereby protecting animals from 2,4,6-trinitrobenzenesulfonic acid solution-induced colitis and intestinal inflammation (88). *A. muciniphila* OMVs are reported to ameliorate DSS-induced colitis using several mechanisms, including restoring the disturbed balance of the gut microbiota, maintaining the integrity of the intestinal barrier, and activating B cells and DCs (45). The IBD-associated genes ATG16L1 and NOD2 are crucial for OMV-mediated activation of colitis protection. ATG16L1 T300A transgenic mice did not exhibit protection from 2,4-dinitrobenzene sulfonic acid-induced colitis. Individuals with Crohn’s disease, a subtype of IBD, typically carry the ATG16L1 major risk variant T300A. This finding suggests a potential target for the early genetic diagnosis of IBD (89).

### Metabolic diseases

The balance of gut microbiota significantly influences host metabolic homeostasis, and BEVs are crucial in this process. A significant increase in OMVs from *Pseudomonas panacis* was observed in a high-fat diet-induced type 2 diabetes mouse model. Subsequent studies confirmed that these OMVs could block the insulin signaling pathway in skeletal muscles and adipose tissues (97). Conversely, *A. muciniphila*-derived OMVs ameliorate high-fat diet-induced obesity via various mechanisms, which include improved intestinal barrier integrity, reduced inflammation, balanced energy, and improved blood parameters (98). This contrasting effect of different BEVs on metabolic homeostasis emphasizes the complex and multifaceted roles of these entities in maintaining host health. It is thought that the characteristics of the parent bacteria determine whether their BEVs are harmful or beneficial, and normal quantity of BEVs could maintain immunological activity while excess amounts would be harmful.

### Gastrointestinal cancer

Numerous reports have highlighted the influence of BEVs on cancer development and metastasis in the gastrointestinal tract. OMVs from *E. coli* MG1655 have been shown to deliver a tRNA fragment termed as Ile-tRF-5X, into human colorectal carcinoma cells (HCT116). This interaction promotes the expression of mitogen-activated protein kinase 3, thereby enhancing cell proliferation (99). *F. nucleatum*, widely recognized as a pathogen that promotes colorectal cancer (CRC) development, utilizes various mechanisms for this process, including OMVs. Proteomic analysis using mass spectrometry revealed an abundance of virulence factors and biologically active proteases present or selectively enriched in these OMVs (100). The specific roles of OMVs in CRC include inducing IL-8 expression (100, 101), which fosters a pro-inflammatory microenvironment favoring tumor growth; and reducing E-cadherin and cadherin-1 gene expression to promote an epithelial-to-mesenchymal transition-like genotype in tumor cells (100, 102), which ultimately promotes the migration and invasion of cancer cells *in vivo* (108).

Conversely studies have explored the potential of BEVs as therapeutic agents for cancer treatment via immunotherapy. Kim et al. found that gram-negative bacterial OMVs specifically targeted and accumulated in tumor tissues of a syngeneic mouse colonic tumor model, subsequently triggering the production of antitumor cytokines CXCL10 and IFN-γ. This indicates that BEVs represent a promising new approach for cancer immunotherapy (103). Additionally, *A. muciniphila* OMVs have been found to enhance programmed cell death protein-1-based immunotherapy of CRC in mouse models. This suggests a potential clinical application of OMVs in improving the efficacy of immunotherapy by targeting programmed cell death protein-1 (45). These varied findings demonstrate the significant and multifaceted roles of BEVs in gastrointestinal cancer progression and potential therapeutic strategies.

## Current challenges and future perspectives

Despite substantial evidence supporting the process of BEVs generation are positive controlled, the regulation of BEVs production and cargo selection remains unclear. Further research is required to elucidate these mechanisms, which will significantly facilitate basic research on BEVs functions in bacteria-bacteria and bacteria-host communication and its translational application. Moreover, the dual role of BEVs in gut health and the pathogenesis of intestinal-related diseases remains unclear. Furthermore, the precise active components of BEVs, their receptors, and the induced signaling pathways in host cells remain unidentified. The investigation of their impact on other intestinal cell types, such as intestinal stromal and neuronal cells is also required.

Considering their ability to penetrate the intestinal barrier and their correlation with various gut diseases, BEVs are potential diagnostic biomarkers for intestinal disorders (106). Their capacity to regulate host immune responses indicates their potential as vaccines against intestinal infections and inflammatory disorders. Preliminary studies have suggested that some BEVs can induce an antitumor immune response and inhibit tumor growth, suggesting their role in cancer immunotherapy (45, 103). Further studies are required to validate these findings and translate them into clinical applications.

Despite the encouraging findings on BEVs, this field of research still remains largely unexplored, and requires more comprehensive investigations a deeper understanding of BEV biogenesis, cargo selection, and their interaction mechanisms with host cells is crucial. With this knowledge, the full potential of BEVs in diagnostics, therapeutics, and vaccine development can be harnessed, thereby opening new frontiers for microbiome-related biomedical applications.

## Author contributions

DS: Conceptualization, Data curation, Funding Acquisition, Writing – original draft, Writing – review & editing. PC: Data curation, Software, Visualization, Writing – original draft. YX: Data curation, Funding acquisition, Writing – review & editing. JS: Conceptualization, Data curation, Funding acquisition, Writing – original draft, Writing – review & editing.
